# SARS-Cov-2 Replication in a Blood–Brain Barrier Model Established with Human Brain Microvascular Endothelial Cells Induces Permeability and Disables ACE2-Dependent Regulation of Bradykinin B1 Receptor

**DOI:** 10.3390/ijms26125540

**Published:** 2025-06-10

**Authors:** Sharton Vinicius Antunes Coelho, Gabriela Lisboa e Souza, Bruno Braz Bezerra, Luan Rocha Lima, Isadora Alonso Correa, Dalziza Victalina de Almeida, Rodrigo Pacheco da Silva-Aguiar, Ana Acácia S. Pinheiro, Pierre Sirois, Celso Caruso-Neves, Luciana Jesus da Costa, Julio Scharfstein, Luciana Barros de Arruda

**Affiliations:** 1 Departamento de Virologia, Instituto de Microbiologia Paulo de Góes, Universidade Federal do Rio de Janeiro, CCS, Bloco I. Av Carlos Chagas Filho, 373, Cidade Universitária, Rio de Janeiro 21941–902, RJ, Brazil; lisboagabriela2002@gmail.com (G.L.e.S.); bezerra@micro.ufrj.br (B.B.B.); luanrocha@micro.ufrj.br (L.R.L.); alonsoc.isadora@micro.ufrj.br (I.A.C.); ljcosta@micro.ufrj.br (L.J.d.C.); 2 Instituto Oswaldo Cruz, Fundação Oswaldo Cruz, Rio de Janeiro 21045-900, RJ, Brazil; dalziza@gmail.com; 3 Instituto de Biofísica Carlos Chagas Filho, Universidade Federal do Rio de Janeiro, Rio de Janeiro 21941–902, RJ, Brazil; rpachecoufrj@gmail.com (R.P.d.S.-A.); acacia@biof.ufrj.br (A.A.S.P.); caruso@biof.ufrj.br (C.C.-N.); jscharf2@gmail.com (J.S.); 4 Department of Microbiology and Immunology, Faculty of Medicine, Universite Laval, 2325 Rue de l’Université, Québec, QC G1V 0A6, Canada; dr.pierre.sirois@gmail.com

**Keywords:** SARS-CoV-2, endothelial cells, blood–brain barrier, ACE2, des-Arg-bradykinin, inflammation

## Abstract

Endothelial dysfunction plays a central role in COVID-19 pathogenesis, by affecting vascular homeostasis and worsening thromboinflammation. This imbalance may contribute to blood–brain barrier (BBB) disruption, which has been reported in long COVID-19 patients with neurological sequelae. The kallikrein–kinin system (KKS) generates bradykinin (BK), a proinflammatory peptide that induces microvascular leakage via B2R. Under inflammatory conditions, BK is converted to Des-Arg-BK (DABK), which activates B1R, a receptor upregulated in inflamed tissues. DABK is degraded by ACE2, the main SARS-CoV-2 receptor; thus, viral binding and ACE2 downregulation may lead to DABK/B1R imbalance. Here, we investigated these interactions using human brain microvascular endothelial cells (HBMECs), as a model of the BBB. Since endothelial cell lines express low levels of ACE2, HBMECs were modified with an ACE2-carrying pseudovirus. SARS-CoV-2 replication was confirmed by RNA, protein expression, and infectious particles release. Infection upregulated cytokines and endothelial permeability, enhancing viral and leukocyte transmigration. Additionally, viral replication impaired ACE2 function in HBMECs, amplifying the response to DABK, increasing nitric oxide (NO) production, and further disrupting endothelial integrity. Our findings reveal a mechanism by which SARS-CoV-2 impacts the BBB and highlights the ACE2/KKS/B1R axis as a potential contributor to long COVID-19 neurological symptoms.

## 1. Introduction

COVID-19 clinical syndromes result from a complex interplay between direct viral cytopathic effects, dysregulated immune responses, and activation of coagulation pathways [1,2]. In severe cases, lung pathology is marked by interstitial edema, fibrin deposition, and inflammatory infiltrates, likely driven by increased vascular permeability [3,4]. Elevated circulating levels of proinflammatory cytokines such as IL-6 and TNF are frequently detected [5], contributing to systemic inflammation. Systemic complications, such as thromboinflammation, have been attributed to dysregulated activation of coagulation pathways, including the extrinsic pathway, driven by elevated expression of tissue factor (TF) by circulating leukocytes and released cellular vesicles [6]. Regarding the contact pathway, other studies have reported increased activation or consumption of factor XII (FXIIa), plasma kallikrein (PKa), and high molecular weight kininogen (HK), all of which are members of the proinflammatory kallikrein–kinin system (KKS) [7,8,9]. Commonly used as biomarkers of thromboinflammation, plasma D-dimers levels helped to identify patients that were at risk of severe COVID-19 [10]. Endothelial activation markers, including von Willebrand factor and thrombomodulin, further reflect vascular injury [11]. More recently, alarmins, including ATP and HMGB1 have been also identified as biomarkers associated with severe or fatal outcomes [12].

Cellular infection is initiated by the binding of the viral spike (S) protein to the angiotensin-converting enzyme 2 (ACE2), the main receptor for SARS-CoV-2 [13]. However, other receptors, such as neuropilin-1 and CD147, have also been implicated in facilitating viral entry [14,15]. The generation of S1 and S2 domains of the S protein depends on processing by host proteases tethered at the cell surfaces of epithelial cells, such as TMPRSS2, or, following endocytic internalization of the virus, by lysosomal cathepsins [13]. Further downstream, viral RNA is released into the cytoplasm [16,17], triggering potent innate immune responses [18].

ACE2 expression has been detected in different human tissues and cell types [19]. However, whereas virus infection in alveolar epithelial cells has been well documented [3,20], evidence of productive virus replication in extrapulmonary sites remains limited and controversial, even though damage or dysfunction had been reported in multiple tissues, including the vasculature [21,22].

It was recently proposed that the neurological syndromes associated with long COVID-19 could result from blood–brain barrier (BBB) disruption [23]. Although the impact of virus replication in BBB function remains unknown, there is precedent that viral RNA persists in the brain of some individuals for several months [24]. These findings suggest that SARS-CoV-2 may reach the CNS by crossing the BBB, either through replication or transcytosis in endothelial cells, or via the extravasation of infected circulating leukocytes, as described for other neuropathogenic viruses [25].

Histopathological analyses, and global single-cell gene and protein expression have shown the presence of ACE2 in the capillary endothelium of the lung, liver, spleen, oral and intestinal mucosa, and brain [19,26]. In addition, viral particles by electron microscopy, as well as histopathological evidence of inflammatory cells and apoptotic signals, were detected in the capillary vessels of post-mortem COVID-19 tissues [27]. However, primary or transformed endothelial cells cultured in vitro have generally shown scanty expression of ACE2, consistent with the fact that they are poorly permissive to SARS-CoV-2 infection [28,29,30]. Nonetheless, when lung (PMEC) or brain endothelial cells (BMECs) were transfected with hACE2, the release of infectious particles was observed [28]. Still, key aspects such as cell activation, permeability alterations, and response to KKS, along with a detailed investigation of SARS-CoV-2 replication in HBMECs, remain underexplored. Additionally, several studies using different models have highlighted the plasticity of endothelial cells, including their capacity to modulate ACE2 expression in response to environmental signals [31]. Therefore, ACE2 expression and the permissiveness of HBMECs could have been underestimated by in vitro culture conditions.

Beyond serving as the surface receptor of SARS-CoV-2, ACE2 has a dual physiological role in vascular homeostasis. This metallopeptidase integrates the renin–angiotensin system (RAS) by converting angiotensin II (AngII) into the anti-inflammatory angiotensin 1– [32,33]. Also, the enzyme plays a pivotal role in the homeostasis of KKS, by degrading des-Arg-bradykinin (DABK) [34]. In the bloodstream, KKS proteolytic cascade involves the activation of plasma kallikreins (PKa), which cleave kininogen (HK), releasing bradykinin (BK). Short-lived BK is either degraded by ACE or induces microvascular leakage by signaling B2R, a G protein-coupled receptor (GPCR) constitutively expressed by the endothelium [35,36]. As the inflammation proceeds, BK is metabolized by kininase I, forming DABK [37]. Differently from the agonist specificity of BK, DABK induces plasma leakage and inflammation by binding to B1R, a GPCR subtype whose expression is strongly upregulated by proinflammatory cytokines [38]. DABK/B1R signaling induces nitric oxide (NO) production, leukocyte recruitment, and increased vascular permeability [36,39,40].

The relevance of ACE2 controlling the DABK/B1R inflammatory axis was evidenced in a mouse model of endotoxin-induced inflammation [41], in which reduced ACE2 activity with impaired DABK degradation was detected. The consequent enhanced B1R signaling resulted in a robust inflammatory response, marked by increased neutrophil infiltration [41]—possibly driven by endothelial activation and permeability. Given the precedent evidence of systemic and pulmonary activation of KKS, as well as increased plasma DABK levels in COVID-19 patients [7,42,43], it is conceivable that ACE2 downregulation by SARS-CoV-2 [44,45] might upregulate the extent of B1R-dependent inflammatory edema during the course of infection.

Here, aiming to understand the effect of SARS-CoV-2 replication in endothelial cells from the BBB, we conducted a systematic investigation of viral replication, using HBMEC, a cell line extensively used as a BBB model in other viral infection systems [46,47,48]. To overcome low ACE2 expression, the cells were transduced with hACE2 pseudovirus. Efficient virus replication occurred only when hACE2 was overexpressed. Productively infected HBMEC-ACE2 cells exhibited increased production of inflammatory cytokines and nitric oxide (NO), as well as enhanced permeability, with higher virus and leukocyte transmigration. Importantly, our studies demonstrated for the first time that SARS-CoV-2 infection impairs ACE2-mediated regulation of the proinflammatory DABK/B1R pathway, raising the possibility that pharmacological targeting of the KKS might protect the BBB from excessive injury caused by SARS-CoV-2.

## 2. Results

### 2.1. Transformed HBMECs Do Not Sustain a SARS-CoV-2 Productive Infection

**Figure 1:**
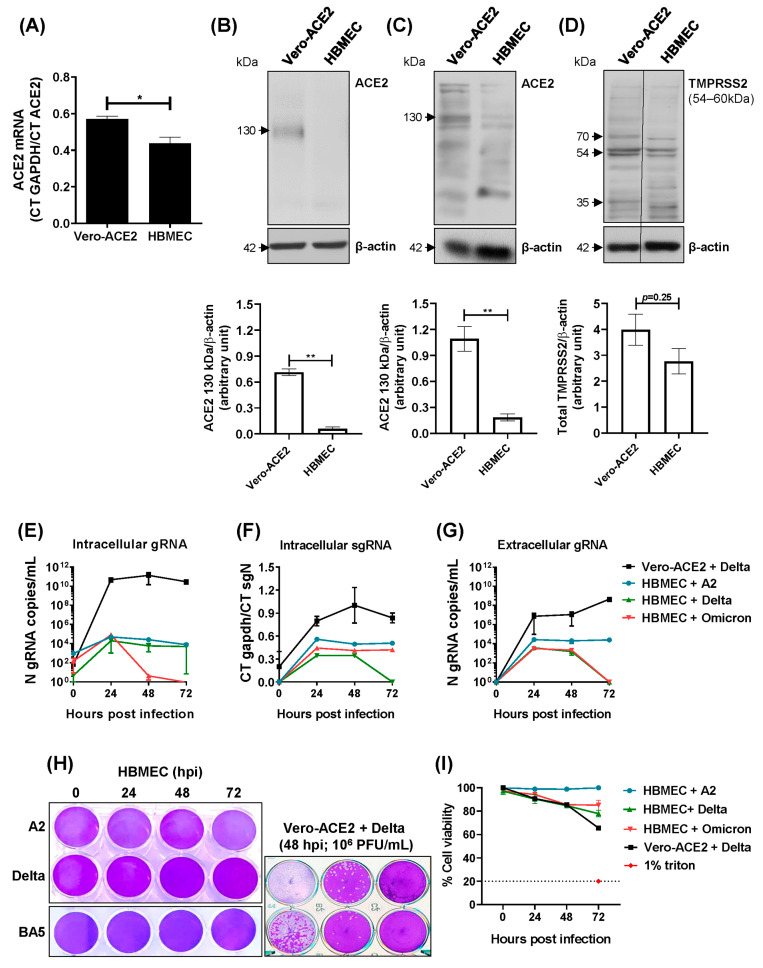
Immortalized HBMECs do not sustain productive SARS-CoV-2 replication. (**A**) Expression of ACE2 mRNA in HBMECs and Vero-ACE2 was measured by RT-qPCR, and gapdh expression was used for normalization. Bars indicate the ratio between Ct GAPDH and ACE2. (**B**–**D**) The expressions of ACE2 (**B**,**C**) and TMPRSS2 (**D**) were evaluated in HBMEC and Vero-ACE2 cells by Western blotting; relative expression in relation to β-actin was measured using ImageJ software version 8.0.2. (**E**–**H**) HBMECs were inoculated with different SARS-CoV-2 lineages, using a MOI of 0.1. Cell lysates (**E**,**F**) and culture medium (**G**,**H**) were harvested at the indicated time points. Viral RNA (**E**–**G**) and infectious virus particles (**H**) were measured by RT-qPCR and plaque assay. (**I**) Cells were infected as in (**E**) and cell viability was measured by LDH released in the culture medium. Data represent the average and SEM from three independent experiments; and were analyzed by unpaired *t* test. * *p* < 0.05; ** *p* < 0.005.

To establish an in vitro BBB model for studying SARS-CoV-2 infection, we investigated whether an HBMEC cell line would support SARS-CoV-2 replication, using a Vero-ACE2 cell line as a gold standard. We first assessed ACE2 receptor expression in HBMECs and found that ACE2 RNA levels were significantly lower than those in Vero-ACE2 cells (Figure 1A). The protein expression was then analyzed by Western blotting using two distinct anti-ACE2 antibodies. ACE2 was undetectable when probed with an antibody specific to the full-length 130KDa protein (Figure 1B), which was reported to localize to the cell surface and contains the catalytic site [49,50]. However, staining with another antibody revealed the presence of ACE2 isoforms, albeit with much weaker immunoreactivity compared to Vero-ACE2 cells (*p* = 0.0036; Figure 1C). The expression of TMPRSS2 was similar between the two cell lines (Figure 1D). HBMECs were then cultured with SARS-CoV-2 lineages A2, Delta, and Omicron BA5.1 and virus replication was assessed by RT-qPCR and plaque assay at different time points. While Vero-ACE2 cells showed increasing viral RNA levels in both cell lysates and culture medium from 24 to 72 hpi, HBMECs displayed only low levels of genomic and subgenomic RNA with no increase over time (Figure 1E–G). Also, no plaque was detected in HBMECs infected with any virus lineage, whereas virus infectious particles were clearly detected in the supernatants of Vero cells (Figure 1H). No cell death was observed in the HBMEC culture until the endpoint of those assays (Figure 1I).

We then argued whether priming with inflammatory stimuli would modulate ACE2 expression and permissiveness to SARS-CoV-2 in HBMECs. HBMEC priming with IFN-β increased ACE2 mRNA, but not protein levels (Figure S1A,B). LPS and TNF treatments did not alter ACE2 expression (Figure S1A,B). In addition, SARS-CoV-2 inoculation in primed cells did not increase the viral RNA copy number in the cell lysates or culture medium (Figure S1C).

### 2.2. HBMECs Overexpressing ACE2 Are Permissive to SARS-CoV-2 Replication

**Figure 2:**
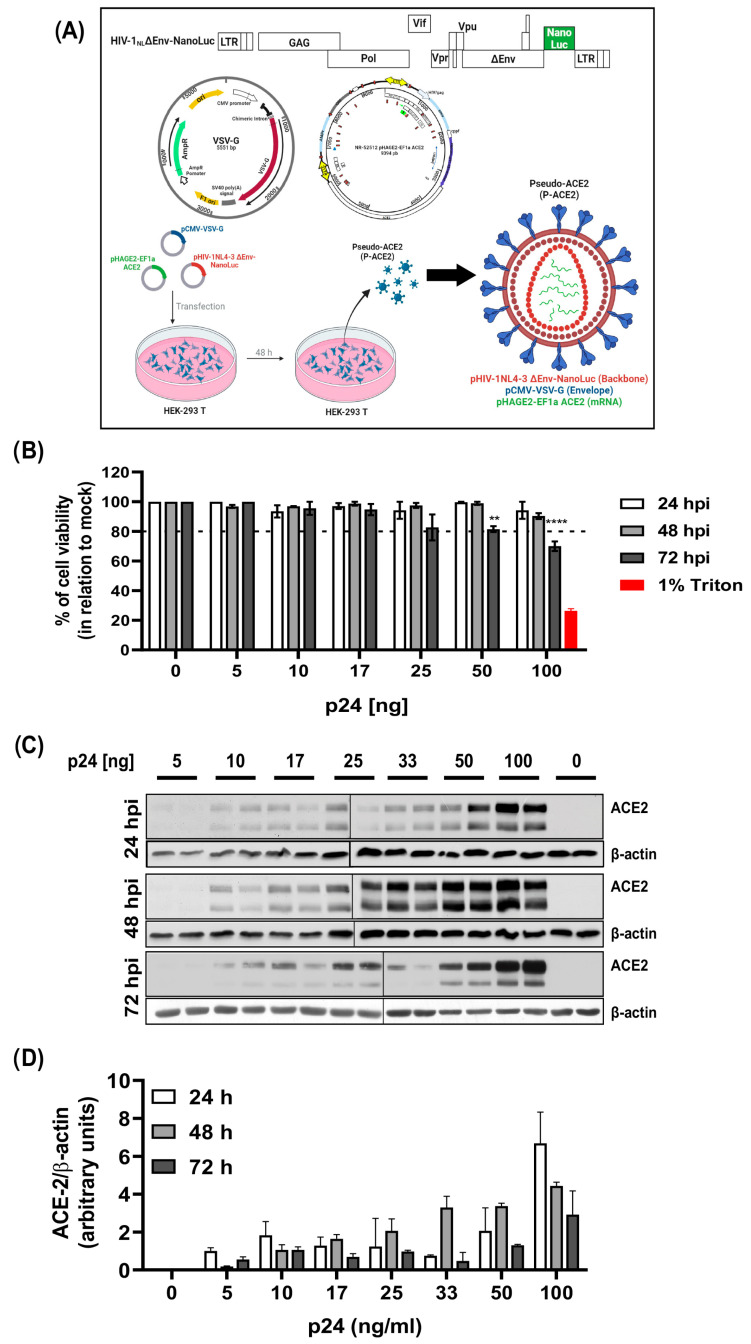
Development of HBMEC line overexpressing ACE2 by transient transduction with ACE2-carrying pseudovirus. (**A**) Schematic representation of the pseudovirus construction (P-ACE2). HEK-293 cells were transfected with pCMV-VSV-G, pHIV-1NL4-3 ΔEnv-NanoLuc, and pHAGE2-EF1a ACE2. After 24 h of transfection, the culture medium was replaced, the cells were cultured for another 24 h, and the supernatant containing pseudovirus was harvested. (**B**) HBMECs were transduced with different concentrations of P-ACE2 (based on p24 ELISA) and the cell viability was measured at the indicated time points by CellTiter Aqueous reagent. Data represent the average and SEM from three independent experiments; and were analyzed by one way ANOVA. ** *p* < 0.005; **** *p* < 0.0001. (**C**,**D**) HBMECs were transduced with P-ACE2 as in (**B**) and the expression of ACE2 was measured by Western blotting at the indicated time points. Representative Western blottings are demonstrated in (**C**) and the ratio between ACE2 and β-actin from 2 different assays is represented in (**D**).

To induce ACE2 expression in HBMECs, we developed a lentivirus-based pseudovirus construction carrying ACE2 mRNA (P-ACE2) (Figure 2A). HBMECs transduced with P-ACE2 were viable (Figure 2B) and showed a remarkable increase in ACE2 protein expression, with a peak at 48 h post-transduction (Figure 2C,D).

**Figure 3:**
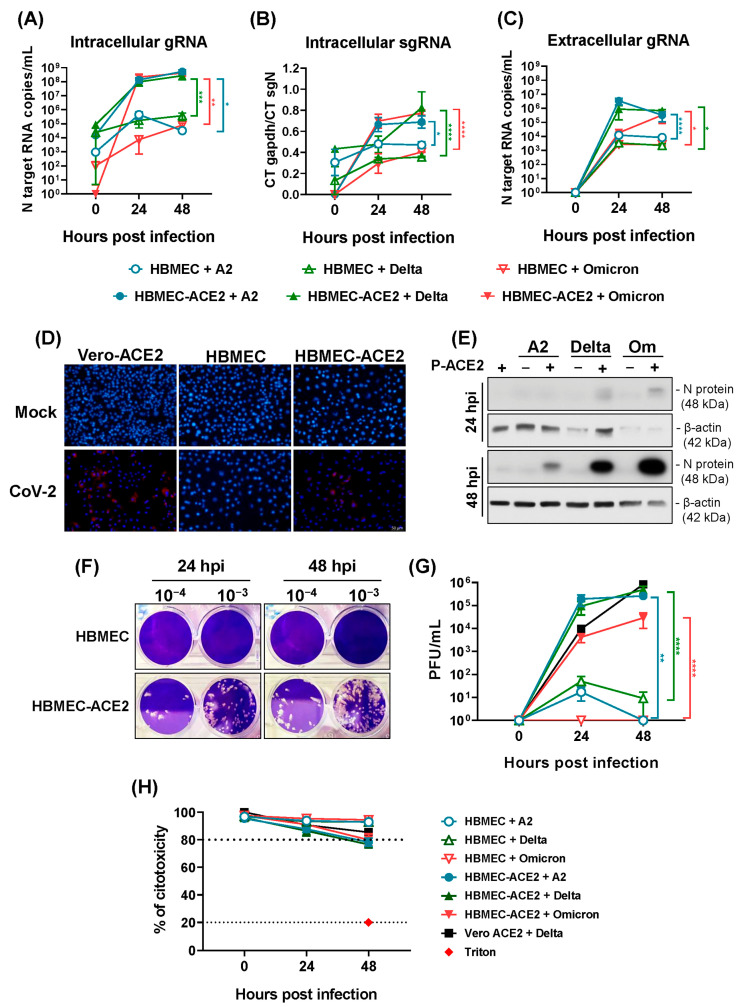
HBMEC-ACE2 is permissive to SARS-CoV-2 replication. HBMECs were transduced or not with P-ACE2 for 48 h and then infected with different lineages of SARS-CoV-2. (**A**,**B**) Cell lysates were harvested at different time points post infection and the concentrations of genomic (gRNA) and subgenomic (sgRNA) virus RNA were measured by RT-qPCR. (**C**) Culture supernatants were harvested at the indicated periods and the released virus RNA was measured by RT-qPCR. (**D**) After 48 h, cells were incubated with J2 antibody and DAPI and the presence of dsRNA-positive cells was evaluated by fluorescence microscope. (**E**) SARS-CoV-2 N protein expression was measured by Western blotting at 24 and 48 hpi. (**F**,**G**) The titer of released infectious particles was measured by plaque assay. A representative assay is shown in (**F**) and the average and SEM from three different experiments are demonstrated in (**G**). Data were analyzed by two-way ANOVA. * *p* < 0.05; ** *p* < 0.005; *** *p* < 0.0005; **** *p* < 0.0001. (**H**) Cell viability was evaluated at different periods post infection by measurement of LDH released in the culture medium.

SARS-CoV-2 replication was then investigated in HBMEC-ACE2, by following the expression of viral RNA, proteins, and the release of infectious particles from 24 to 72 hpi. The concentrations of intracellular genomic and subgenomic RNA copies were higher in HBMEC-ACE2 compared to HBMEC, from 24 hpi, and further increased at 48 hpi (*p* < 0.05) (Figure 3A,B). J2 antibody staining and immunofluorescence analysis revealed dsRNA-positive cells only in the culture of SARS-CoV-2-infected HBMEC-ACE2, but not in the WT ones (Figure 3D). The viral nucleocapsid protein (N) from all lineages was also exclusively detected in HBMEC-ACE2 cells (Figure 3E). In addition, a significant difference in the amount of released viral RNA was observed at 24 and 48 hpi in HBMEC-ACE2 cells compared to HBMECs (*p* < 0.0001) (Figure 3C). Importantly, HBMEC-ACE2 released infectious virus particles reaching more than 10^5^ PFU/mL at 48 hpi (Figure 3F,G). Still, none of the experimental settings resulted in significant cell death (Figure 3H and Figure S2).

### 2.3. Production of Inflammatory Mediators by HBMEC-ACE2 Infected with SARS-CoV-2

**Figure 4:**
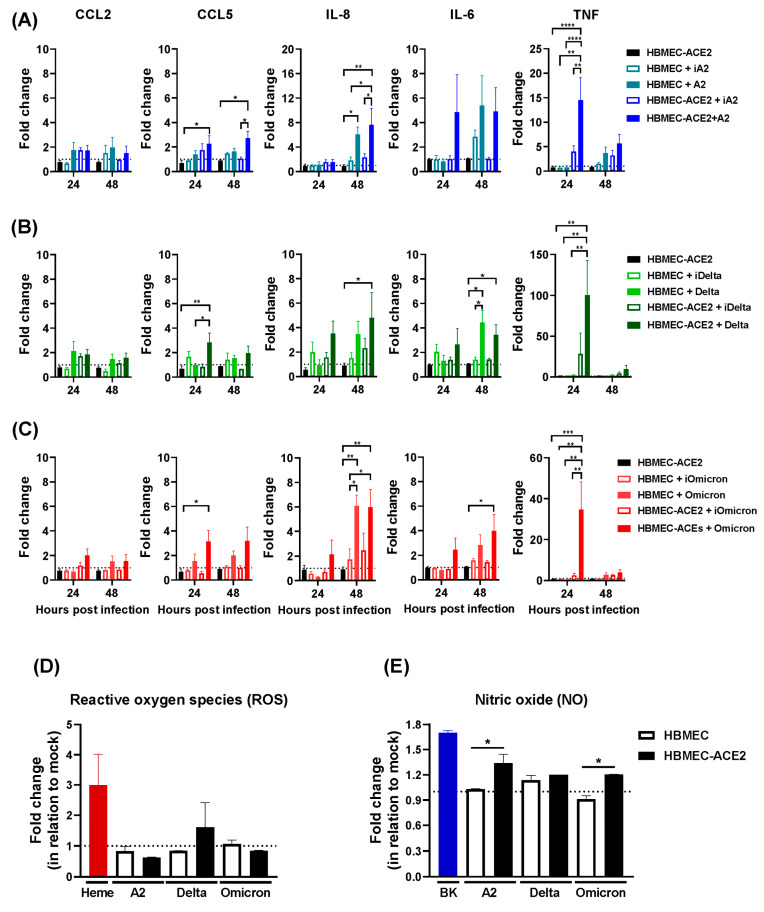
SARS-CoV-2 replication-dependent and -independent expression of inflammatory cytokines. HBMECs or HBMEC-ACE2 were inoculated with native (A2, Delta, Omicron) or inactivated (iA2, iDelta, iOmicron) SARS-CoV-2 from the lineages A2 (**A**), Delta (**B**), or Omicron BA1 (**C**). Cell lysates were harvested at 24 and 48 hpi, and the concentrations of mRNA corresponding to the indicated chemokines (CCL2, CCL5, IL-8) and cytokines (IL-6, TNF) were measured by RT-qPCR. Data represent the average and SEM from three independent experiments; and were analyzed by two-way ANOVA. * *p* < 0.05; ** *p* < 0.005; *** *p* < 0.0005; **** *p* < 0.0001. (**D**) After 24 hpi, cells were incubated with CM-H2DCFDA fluorescent probe, and the generation of reactive oxygen species (ROS) was evaluated by spectrophotometry; Heme was added as a positive control. (**E**) After 24 hpi, cells were incubated with DAF-FM diacetate fluorescent probe and nitric oxide (NO) production was evaluated by spectrophotometry; bradykinin (BK) was added to the cultures, as a positive control. The bars indicate the average and SEM from three independent experiments and data were analyzed by one way ANOVA, followed by Tukey’s multiple comparison test; * *p* < 0.05.

We subsequently assessed the impact of SARS-CoV-2 replication on molecular parameters implicated in vascular function. SARS-CoV-2 infection significantly increased CCL5 and IL-8 mRNA levels, but not CCL2. CCL5 upregulation occurred only in HBMEC-ACE2 cells infected with native viruses, while IL-8 expression was elevated in both transduced and non-transduced cells. TNF levels were also significantly higher in infected HBMEC-ACE2, but not in WT cells or cultures exposed to inactivated viruses. IL-6 was upregulated in both cell lines upon infection (Figure 4A–C). To further investigate the impact of virus entry on endothelial cell activation, the cells were pretreated with the TMPRSS2 inhibitor camostat mesylate. Camostat was not toxic to HBMEC, and, corroborating the previous results, pretreating HBMEC with the inhibitor reduced both virus replication and the expression of CCL5 and TNF, but not of IL-8 (Figure S3).

SARS-CoV-2 infection did not affect the generation of ROS (Figure 4D). However, enhanced NO production was detected in infected HBMEC-ACE2, but not in WT cells (Figure 4E), showing its dependence on virus replication.

### 2.4. Infection of HBMEC-ACE2 with SARS-CoV-2 Induces Endothelial Permeability, with Virus and Mononuclear Cells Crossing

**Figure 5:**
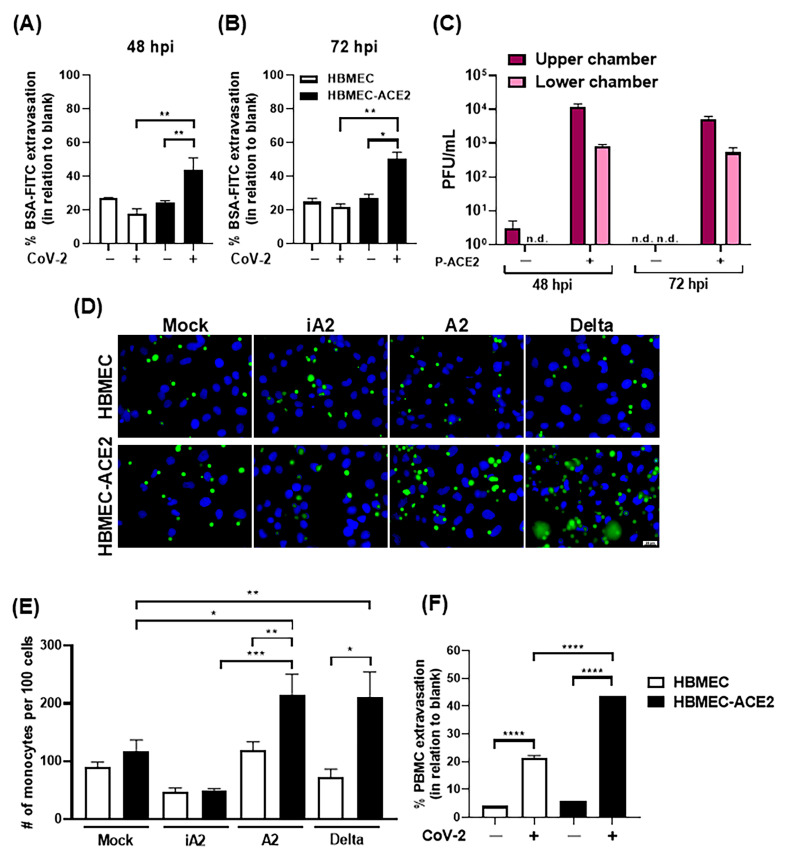
SARS-CoV-2 replication in HBMEC-ACE2 induces permeability, with crossing of viruses and mononuclear cells. HBMECs were transduced or not with P-ACE2 for 48 h and then infected with SARS-CoV-2. At 48 (**A**) and 72 hpi (**B**), the cells were incubated with BSA-FITC for 1 h and the amount of protein detected in the lower chamber was measured by fluorimetry. The bars represent the average and SEM from three independent experiments and the data were analyzed by unpaired *t* test. (**C**) At the indicated time points, culture medium from the upper and lower transwell chambers was harvested and the virus titers were measured by plaque assay. n.d.—not detected. (**D**,**E**) At 48 hpi, cells were incubated with CFSE-stained monocytes for 30 min and the number of adhered cells was analyzed by confocal fluorescence microscopy and measured using ImageJ software. Representative images are shown in (**D**) and the average and SEM from five independent experiments are indicated in (**E**). (**F**) HBMECs or HBMECs-ACE2 were cultured in a transwell system and, after 48 hpi, cells were incubated with PBMC. The percentage of PBMCs in the lower transwell chamber was calculated in relation to the wells with no cells (blank-100%). Data are representative of two independent assays. The data were analyzed by one way ANOVA, followed by Tukey’s multiple comparison test; * *p* < 0.05; ** *p* < 0.005; *** *p* < 0.0005; **** *p* < 0.0001.

Endothelial permeability of infected cells was then assessed in a transwell system. A significant increase in BSA-FITC extravasation was detected in HBMEC-ACE2 cultures at 48 and 72 hpi (Figure 5A,B). Additionally, infectious virus particles were detected in the lower transwell chamber, indicating virus crosses through the endothelial monolayer (Figure 5C).

Subsequently, HBMEC or HBMEC-ACE2 were infected with SARS-CoV-2 and incubated with CFSE-stained primary human monocytes. Enhanced cellular adhesion was detected in HBMEC-ACE2 infected with native, but not inactivated virus (Figure 5D,E). In another setting, HBMECs were incubated with primary PBMCs in a transwell system, and the number of PBMCs in the lower chamber was counted. A significantly higher number of PBMCs was detected in HBMEC-ACE2 cultures infected with SARS-CoV-2, compared to WT and non-infected cells (Figure 5F), indicating that SARS-CoV-2 replication promoted increased monocyte adhesion and transmigration.

### 2.5. SARS-CoV-2 Infection Modulates ACE2-Dependent Regulation of DABK/B1R Pathway

**Figure 6:**
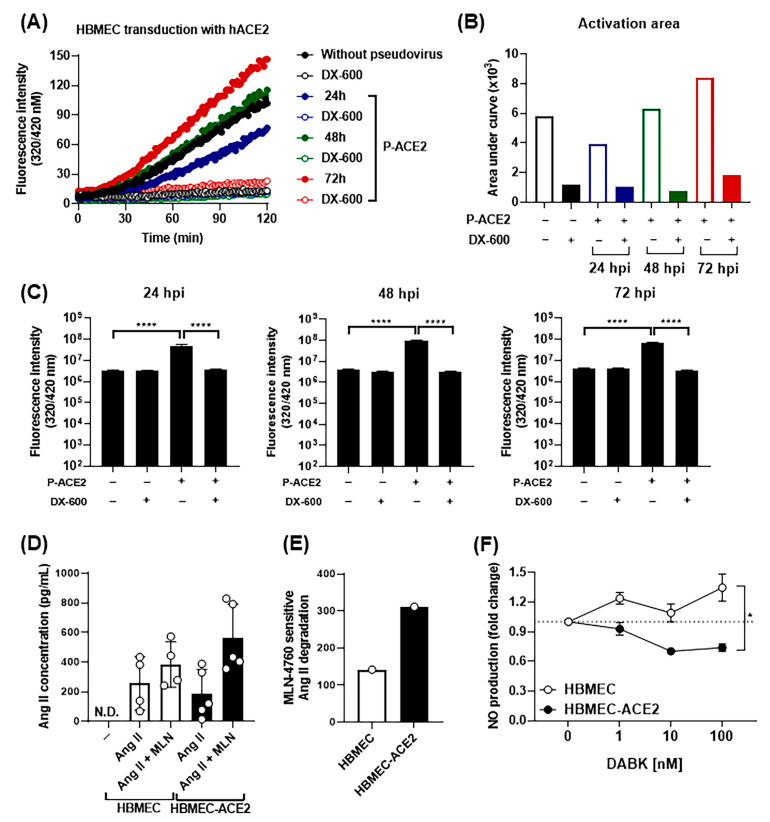
ACE2 transduction in HBMECs downmodulates RAS and KKS. (**A**,**B**) Cell lysates obtained from HBMECs or HBMEC-ACE2 were incubated with the ACE2 fluorogenic substrate, in the presence or not of DX600. Activation of ACE2 was evaluated by measuring fluorescence emission due to substrate hydrolysis for 2 h. A representative curve obtained after spectrofluorimetric analysis is shown in (**A**), and the activation area, determined by subtracting the curve area obtained from lysates incubated or not with DX-600 is shown in (**B**). (**C**) After 24, 48, or 72 h post P-ACE2 transduction, intact culture cells were incubated with the ACE2 substrate for 2 h, and fluorescence emission was measured. (**D**,**E**) HBMEC or HBMEC-ACE2 were incubated with exogenous angiotensin II (AngII), in the presence or absence of MLN-4760. After 6 h, AngII concentration in the culture medium was measured by ELISA (**D**) and MLN-sensitive Ang II degradation (**E**) was calculated as the ratio between cultures with and without the ACE2 inhibitor. (**F**) HBMEC or HBMEC-ACE2 were incubated with increasing concentrations of DABK, and the production of nitric oxide (NO) was measured by spectrophotometry. The data were analyzed by one way ANOVA, followed by Tukey’s multiple comparison test; * *p* < 0.05; **** *p* < 0.0001; N.D.—not detected.

After showing that HBMECs-ACE2 are susceptible to SARS-CoV-2 infection, we sought to determine whether the transduced ACE2 was enzymatically active in whole cell lysates by measuring the hydrolysis of an intramolecularly quenched fluorogenic substrate, in the presence or absence of the ACE2 inhibitor DX-600. Our results (Figure 6A,B) did not show obvious differences between cell lysates of both cell lines, most likely due to similar levels of intracellular isoforms of ACE2. In an effort to detect enzymatically active ACE2 at the surface of these cultures, the fluorogenic substrate was added to intact cell cultures. Under these conditions, increased hydrolysis was only observed in the HBMEC-ACE2 culture medium, and the reaction was significantly reduced by DX-600 (Figure 6C). Having concluded that ACE2 was enzymatically active at the surface of transduced cells, we next compared the ability of both cell lines to degrade Ang II. To this end, HBMECs or HBMEC-ACE2 were incubated with synthetic Ang II, with or without the specific ACE2 inhibitor MLN-4760. Levels of Ang II in the culture medium were determined by ELISA (Figure 6D), and the ratio of Ang II degradation in the presence and absence of the ACE2 inhibitor was calculated. In line with our predictions, ACE2-mediated degradation of Ang II was more accentuated in HBMEC-ACE2 as compared to HBMECs (Figure 6E). We previously showed that the cell line HBMECs constitutively express B1R [51]. We took advantage of B1R expression to monitor DABK-induced NO production in both cell cultures. As expected, HBMECs produced NO in response to DABK, while HBMEC-ACE2 did not (Figure 6F), substantiating the hypothesis that ACE2 transduction translated into DABK degradation.

**Figure 7:**
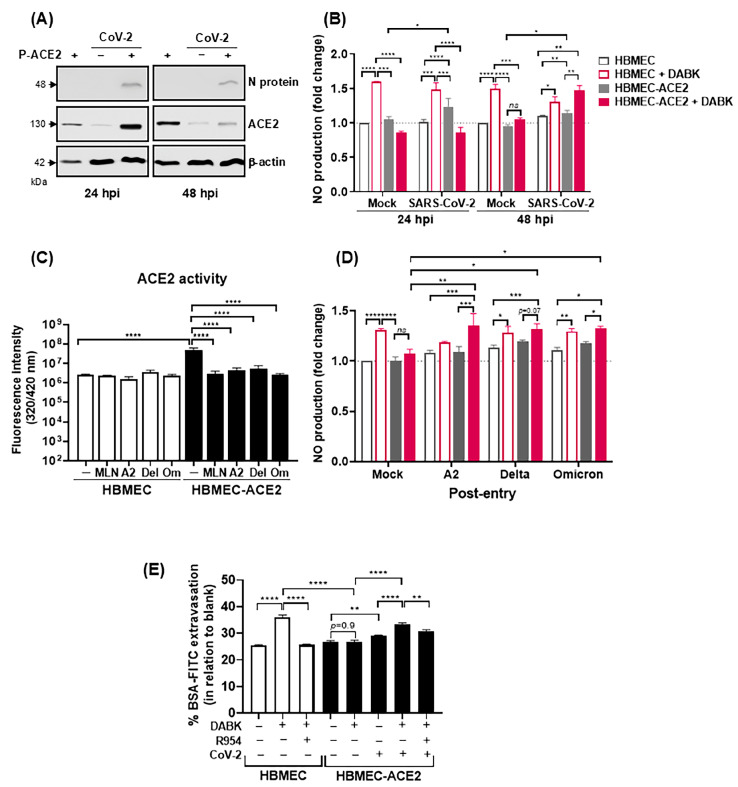
ACE2 downregulation by SARS-CoV-2 promotes an increase in DABK-mediated NO production and endothelial permeability. HBMECs were transduced or not with P-ACE2 for 48 h and then infected with SARS-CoV-2. (**A**) The expression of ACE2 was evaluated by Western blotting at 24 and 48 hpi. (**B**) After 24 or 48 hpi, the cells were incubated with des-Arg-BK (DABK) for 30 min and NO production was evaluated by spectrophotometry. (**C**) Cells were inoculated with the indicated SARS-CoV-2 lineages at an MOI of 10. At 1 h post entry, the fluorogenic ACE2 substrate was added, in the presence or not of MLN-4760. Enzymatic activity was measured by fluorescence emission in the culture medium. (**D**) Cells were infected as in (**C**) and then treated with DABK. NO production was measured as previously described. (**E**) HBMECs and HBMEC-ACE2 were cultured in a transwell system and infected or not with SARS-CoV-2 (delta lineage) for 48 h. Then, the cells were stimulated with DABK, in the presence or absence of R954. After 16 h stimulation, the cultures were incubated with BSA-FITC and permeability was measured by fluorimetry in the lower transwell chamber; data are representative of two independent experiments; the data were analyzed by one way ANOVA, followed by Tukey’s multiple comparison test; ns (not significant); * *p* < 0.05; ** *p* < 0.005; *** *p* < 0.0005; **** *p* < 0.0001.

SARS-CoV and SARS-CoV-2 infection was previously shown to downmodulate ACE2 [44,45]; therefore, we assessed whether virus replication would modulate the response to DABK. ACE2 expression in the transduced cells was confirmed by Western blotting, and the infection significantly decreased ACE2 at 48 hpi (but not at 24 hpi), in comparison to mock-treated cells (Figure 7A). Corroborating the previous result, functional assays confirmed that the addition of DABK to mock-treated HBMECs, but not HBMEC-ACE2, induced NO production (Figure 7B). In contrast, at 48 hpi, stimulation with DABK increased NO levels in both HBMEC and HBMEC-ACE2, suggesting that virus replication overcame ACE2-mediated downregulation of DABK.

Since ACE2 downregulation is more evident shortly after virus entry [44,45], we assessed the enzyme activity 2 h after SARS-CoV-2 inoculation. Confirming the previous data, the hydrolysis of the ACE2 substrate was significantly increased in HBMEC-ACE2 cultures, compared to control HBMECs (Figure 7C). Notably, SARS-CoV-2 inoculation reduced ACE2-mediated hydrolysis to control levels, comparable to cells treated with the ACE2 inhibitor MLN-4760. Additionally, DABK-induced NO production was increased in SARS-CoV-2-infected HBMEC-ACE2, regardless of the virus lineage (Figure 7D).

Finally, we addressed whether virus infection modulated DABK-evoked endothelial permeability. HBMECs or HBMEC-ACE2 were cultured with DABK, with or without the B1R antagonist R954, and the permeability to BSA-FITC was assessed in a transwell system. As predicted, the addition of DABK to HBMECs increased FITC-BSA leakage, but this response was blocked by R954 (Figure 7E). FITC-BSA extravasation was not detected in mock-treated HBMEC-ACE2 stimulated with DABK. Importantly, SARS-CoV-2 infection induced BSA-FITC extravasation and the permeability of the endothelial barrier was significantly enhanced upon addition of DABK to the cultures. Furthermore, DABK-mediated permeability in infected HBMEC-ACE2 was significantly inhibited by the B1R antagonist. Collectively, these findings suggest that SARS-CoV-2 infection inhibits the anti-inflammatory activity of ACE2, rendering the BBB increasingly permeable as a result of hyperactivation of the DABK/B1R pathway.

## 3. Discussion

In the present study, we used a model of HBMECs overexpressing hACE2 to scrutinize aspects of brain endothelial cell function affected by SARS-CoV-2 replication and its impact on disease pathogenesis. To our knowledge, this is the first study to evaluate in detail the various steps of viral biosynthesis, from adsorption to the release of infectious particles, providing solid information about HBMEC permissiveness to SARS-CoV-2 under in vitro conditions. We showed that ACE2 is essential for virus replication in HBMECs and that downregulation of its activity by SARS-CoV-2 allows increased availability of the vasoactive DABK, resulting in endothelial permeability. This model is particularly relevant for investigating acute and prolonged neurological syndromes, as HBMEC culture represents a simplified model of the BBB, the disruption of which was recently associated with cognitive impairment in long COVID-19 patients [23].

Previous reports investigating endothelial cells infection in vitro demonstrated that primary cells from the lungs, heart, kidney, and brain, as well as immortalized HBMECs, present low ACE2 expression and inefficient SARS-CoV-2 replication [28,30,52,53]. On the other hand, ex vivo models, based on histopathological, single-cell gene, or protein expression analyzes, evidenced ACE2 expression in endothelial cells from human lung, liver, spleen, oral and intestinal mucosa, and brain [19,26], as well as in rodent experimental models [54].

Endothelial cell plasticity and differential responses under resting or inflammatory conditions affect various vascular functions [55,56], potentially impacting ACE2 expression. A previous study found that ACE2 was undetectable in isolated brain cells, but its expression significantly increased in 3D vessel models endothelialized with HUVEC or HBMECs under shear stress [31]. Therefore, ACE2 expression and the permissiveness of HBMECs might have been underestimated by in vitro culture conditions.

Our study, using HBMECs overexpressing ACE2, addresses this issue, showing increased virus adsorption, expression of the N protein and dsRNA, and increasing genomic and subgenomic RNA expression in the cell lysates and culture medium, along with infectious particles release. All these assays indicated that HBMEC-ACE2 were fully permissive to SARS-CoV-2 replication, representing a relevant model to investigate the impact of virus infection on endothelial cell function.

Regarding the endothelium associated with the central nervous system, virus-like structures [57] and expression of the spike gene [58] were observed in vascular structures upon histopathological analysis of human post-mortem tissues. A Viral genome was also detected in the brain vascular endothelial cells in hamster and hACE2 transgenic mouse models [54], raising the possibility that endothelial cells were infected by SARS-CoV-2 in vivo. Neurological damage and neuropsychiatric effects are frequently observed in both mild and severe COVID-19 patients, often persisting as long COVID-19 sequelae [23,59]. While BBB damage has been observed [23], its role in virus entry into the CNS remains unclear. Positive PCR results, dsRNA, and S staining in brain autopsies suggest virus invasion [59,60], and viral RNA has been detected in the brain later, after the acute symptoms [24]. However, some studies did not detect viruses in the brains nor in the cerebrospinal fluid (CSF) but evidenced tissue injury and neuroinflammation [61,62].

Understanding the biological effects of SARS-CoV-2 infection in BBB cells is crucial for addressing both viral invasion and the pathological impact of activated brain endothelial cells. SARS-CoV-2 replication in HBMEC-ACE2 stimulated the expression of chemokines and inflammatory cytokines. The production of CCL5 and TNF was clearly dependent on virus replication, but IL-6 and IL-8 were also induced in non-transduced HBMECs, which may have resulted from S stimulation, as previously reported by others [63,64]. Importantly, SARS-CoV-2 infection increased endothelial permeability, promoting virus crossing and mononuclear cells adhesion and transmigration, dependent on ACE2 expression and productive replication. Accordingly, vascular damage and leakage were evidenced in the brains of COVID-19 patients, along with the presence of monocytes and lymphocytes in perivascular regions [62].

A major finding of our study is that SARS-CoV-2 binding and/or replication resulted in lower ACE2 enzymatic function and increased cell response to the vasoactive peptide DABK. KKS is a major controller of vascular tonus and permeability, and it is tightly regulated by tissue and circulating degrading enzymes to maintain homeostasis. These features represent intrinsic technical challenges in measuring the concentration of KKS elements and assessing their function ex vivo or in vitro. Nonetheless, KKS activation was previously reported, with increased consumption of PKa and HK in patients’ serum/plasma [7,8]. Elevated levels of tissue kallikrein and BK metabolites were also reported in the BALF of COVID-19 patients [42] and S and M proteins were shown to bind HK and FXII, generating BK [65]. Increased expression of both BK receptors had also been observed in circulating and BALF cells from COVID-19 patients, but their functional modulation was not addressed [7,66]. Since ACE2 carboxypeptidase activity promotes DABK degradation, its downregulation by virus infection [45] presumes increased availability of DABK and B1R signaling. Indeed, we showed that HBMEC-ACE2 poorly responds to exogenous DABK, as assessed by NO production and endothelial permeability assays. However, if cells were previously infected with SARS-CoV-2, NO production was restored, and permeability was significantly enhanced.

A limitation of our study is the use of a simplified in vitro BBB model that includes only HBMECs, and did not consider the effect of infection in the whole neurovascular unit, including pericytes, astrocytes, and microglia. ACE2 expression was demonstrated in pericytes [67], and infection of these cells and of astrocytes have been suggested [59,67]. Also, evidence of astrogliosis and microgliosis was detected in postmortem samples [68]. Although the KKS has not yet been explored in these cells, their infection or activation, in conjunction with endothelial cells, may further compromise BBB integrity and exacerbate neuroinflammation. This response could be further amplified by endothelial activation with production of TNF, as observed in our model, which may upregulate B1R expression in neighboring cells and enhance KKS activation within the neurovascular unit. Additionally, post-mortem lung biopsies from COVID-19 patients revealed high density of mast cells in alveolar septa and perivascular spaces [69]. It is tempting to speculate that mast cell infiltration and activation in the CNS—leading to the release of polyphosphates, which are known to activate plasma contact factors [70], may contribute to local activation of KKS. This, in turn, could enhance DABK generation and B1R signaling, thereby further amplifying vascular leakage and inflammatory damage. Therefore, the development of more complex multicellular or in vivo models will be essential to fully understand how SARS-CoV-2-mediated dysregulation of the ACE2/KKS/B1R axis contributes to BBB disruption and neurological manifestations of COVID-19.

## 4. Materials and Methods

### 4.1. Cells and Viruses

HBMEC [71] was kindly provided by Dr Dennis J. Grab (Uniformed Services University of the Health Sciences, Bethesda, MD, USA); HEK-293 T cells and Vero E6 cells expressing Transmembrane Protease Serine 2 and Human Angiotensin-Converting Enzyme 2 (Vero E6-TMPRSS2-T2A-ACE2 cells (Vero-ACE2); NR-54970) were kindly provided by Dr. Amilcar Tanuri (Institute of Biology, UFRJ). All cell lines were cultured in DMEM high Glucose (DMEM; Thermo Fisher Scientific Inc., Pittsburgh, PA, USA) and supplemented with 10% fetal bovine serum (FBS).

SARS-CoV-2 A2 (GISAID: 528539), Delta (GISAID: 8433592), and Omicron BA5.1 (GISAID: 8433887) were isolated from symptomatic individuals attending the Center for Managing and Studies of Emerging and Re-emerging Infectious Diseases (Needier, UFRJ). This study was approved by the National Committee of Research Ethics (CAAE-30161620.0.1001.5257). All SARS-CoV-2 strains were sequenced as previously described [72]. Virus stock production and titration were performed in Vero E6, as previously described [73]. Inactivated viruses were obtained by heating the stock at 56 °C for 60 min, and culture medium obtained from non-infected Vero E6 cells was used as mock control.

### 4.2. Pseudovirus Construction and Transduction in HBMECs

The plasmids pCMV-VSV-G (RRID:Addgene_8454) [74], pHIV-1NL4-3ΔEnv-NanoLuc, and pHAGE2-EF1a ACE-2 (BEI Resources NR-52512) were used to form a pseudovirus carrying the human ACE2 (hACE2). The plasmids were amplified in *E. coli* (Max Efficiency stbl2) and purified with Qiagen Plasmid Maxi Kit, according to the manufacturer’s protocols (Qiagen, Hilden, Germany). HEK293-T cells were transfected with the referred plasmids, using calcium chloride, at room temperature, for 40 min. The cultures were incubated for 48 h. The supernatants were harvested, centrifuged, and filtered to obtain the purified pseudovirus P-ACE2. The quantification of the produced pseudovirus stocks was determined by measuring the p24 levels in the supernatants by ELISA (ZeptoMetrix^®^ RETROtek HIV-1 p24 Antigen ELISA, Buffalo, NY, USA), following the manufacturer’s instructions.

To obtain HBMEC overexpressing hACE2 (HBMEC-ACE2), the cells were infected with different P-ACE2 concentrations and incubated for 24, 48, or 72 h, and hACE2 expression was evaluated by Western blotting.

### 4.3. HBMECs Infection with SARS-CoV-2

HBMEC or HBMEC-ACE2 were inoculated with SARS-CoV-2 (A2, Delta, or Omicron BA5), at an MOI of 0.1. As a negative control, cells were incubated with mock or inactivated viruses. After 1 h of virus adsorption, the cells were washed and cultured with complete medium for the indicated time points. Cell lysates and supernatants were harvested and stored at −80 °C. In some experiments, the cells were pretreated with 1 µM of camostat mesylate (Sigma-Aldrich, St. Louis, MO, USA) for 2 h at 37 °C, and then infected, as described.

### 4.4. Analysis of Virus Replication, hACE2 and Cytokine Expression by RT-qPCR

HBMECs or HBMEC-ACE2 were cultured with mock or inactivated viruses, or infected with native SARS-CoV-2, as described. In some experiments, the cells were previously stimulated with LPS for 4 h (1 μg/mL; Merck, Burlington, MA, USA), or IFN-β for 24 h (1000 U/mL; PeproTech, Cranbury, NJ, USA), or TNF for 4 h (10 ng/mL; Merck, Darmstadt, Germany). RNA samples were isolated from the cell lysates and supernatants using TRIzol reagent, and first-strand cDNA was synthesized using a High-Capacity cDNA Archive kit (Thermo Fisher Scientific Inc., Pittsburgh, PA, USA), according to the manufacturer’s instructions. The obtained cDNA was subjected to qPCR reaction using the primers and probe described in Table 1. Analysis of genomic viral RNA was performed using SARS-CoV-2 N2 primers (synthesized as primetime chemistry by Integrated DNA Technologies (IDT) according to sequences obtained from the US CDC) and conducted in AriaMX Real-Time PCR System (Agilent, version 3.1.1812.0301) using the TaqMan Mix (Thermo Fisher Scientific Inc.), as previously described [73]. Analysis of intracellular subgenomic viral RNA was performed using specific primers for N gene subgenomic product and a probe for N gene, with the same cycling parameters. Ace2, Ccl2, Ccl5, Il8, Tnf, Il6, and housekeeping Gapdh expression were measured using Power SYBR Green PCR master mix reagent (Thermo Fisher Scientific Inc.) and the following cycling parameters: 95 °C for 2 min, and 40 cycles composed by 1 cycle consisting of denaturation (95 °C, 15 s) and primer annealing/extension (60 °C, 1 min); finally, samples were subjected to a melting curve analysis to eliminate primer dimers: 95 °C for 15 s, 60 °C for 1 min, and 95 °C for 15 s. The comparative CT method (2^−ΔΔCt^) was used to quantify gene expression levels, using GAPDH for normalization.

### 4.5. Evaluation of Protein Expression by Western Blotting

After the indicated time points, the cells were harvested and protein extracts were subjected to SDS-PAGE, followed by transfer to a nitrocellulose membrane. The total protein content was normalized using the Bradford assay, and 50 μg of protein were loaded onto the gel. Membranes were blocked using 5% nonfat dried milk diluted in Tris Buffered Saline with 0.1% Tween 20, and incubated, overnight at 4 °C, with the following antibodies: anti-ACE2 (Ref.: Ab272500, Abcam.—Figure 1C and Figure S1B; or Ref.: Ab108252, Abcam—Figure 1B, Figure 2C and Figure 7A), anti-SARS-CoV-2 S protein (Ref.: 569965, Cell Signaling Technology, Inc., Danvers, MA, USA), anti-Nucleocapsid (Ref.: 26369, Cell Signaling Technology), anti-TMPRSS2 (Proteintech, Chicago, IL, USA; Ref: 14437-1-AP), and anti-β-actin (Ref.: A2228, Sigma-Aldrich). Then, the membranes were incubated with HRP-conjugated secondary antibodies (Santa Cruz Biotechnology, Dallas, TX, USA). Super Signal West Pico chemiluminescent substrate (Thermo Fisher Scientific Inc.) was used for protein detection following the manufacturer’s instructions. The ratio of the protein of interest and β-actin was determined using ImageJ software, version 8.0.2.

### 4.6. Cell Viability Assay

Cell viability was assessed using CellTiter Aqueous One Solution (Promega, Madison, WI, USA) and LDH release in the supernatants (Promega, Madison, WI, USA—ref.: G1780), according to the manufacturer’s protocols. Absorbance readings were taken using a spectrophotometer (GloMax^®^-Promega, Madison, WI, USA). A 1% Triton X-100 solution was used as a positive control for cell death in both assays.

### 4.7. Evaluation of dsRNA Staining by Immunofluorescence

HBMECs or HBMEC-ACE2 were infected with SARS-CoV-2, as described. At 48 hpi, the cells were fixed with 4% formaldehyde, permeabilized with 0.1% of saponin, and stained with J2 antibody (0.4 μg/mL in 3% BSA, Ref.: 76651, Cell Signaling Technology), for 2 h, followed by AlexaFluor594-conjugated anti-mouse IgG (1 μg/mL, Ref.: A32742, Thermo Fisher Scientific Inc). DAPI was used to stain cells’ nuclei. The images were obtained by fluorescence microscopy, using OLYMPUS IX81 equipment.

### 4.8. Quantification of Nitric Oxide (NO) and Reactive Oxygen Species (ROS)

HBMECs or HBMEC-ACE2 were infected with the SARS-CoV-2 lineages, as described. At 24 hpi, the cells were washed and incubated with DAF-FM diacetate (4-Amino-5-Methylamino-2′,7′-Difluorofluorescein Diacetate; Thermo Fisher Scientific Inc.) or CM-H2DCFDA (Thermo Fisher Scientific Inc.) for 40 min in PBS. Subsequently, the cells were washed, and NO or ROS fluorescence was measured using a SpectraMax i3 spectrophotometer (Molecular Devices, Lagerhausstrasse, Austria). As positive controls for NO and ROS production, respectively, the cells were cultured with BK (1 µM) or Heme (30 µM). In some experiments, HBMEC or HBMEC-ACE2 were incubated with increasing concentrations of DABK (1–100 nM) for 30 min before DAF-FM staining.

### 4.9. Assessment of ACE2 Enzymatic Activity

ACE2 enzymatic activity in whole cell lysates was assessed by incubating total proteins with 5 µM of the fluorogenic quenched ACE2 substrate (MCA-Ala-Pro-Lys(Dnp)-COOH; Aminotech, Sorocaba, SP, Brazil) [75], with or without the ACE2 inhibitor DX-600 (10 µM; Aminotech) [76]. Fluorescence kinetics were performed at 37 °C for 120 min. The activation area was calculated by subtracting the curves obtained from 120 min spectrofluorimetric analysis (O.D.) from lysates incubated or not with DX-600. Evaluation of the enzymatic activity of surface ACE2 was performed by adding the substrate and inhibitor to intact cell cultures for 2 h. Fluorescence emission was measured as described. In some assays, HBMECs or HBMEC-ACE2 were previously infected with SARS-CoV-2, at a MOI of 10, for 2 h at 37 °C.

### 4.10. Ethical Statement and Isolation of PBMC and Human Primary Monocytes

Blood samples (fresh buffy coats) from healthy donors were obtained from the Hemotherapy Service at the Hospital Universitario Clementino Fraga Filho (HUCFF) of Universidade Federal do Rio de Janeiro (UFRJ). The study protocol was approved by the Experimental Ethics Committee of UFRJ (protocol number 105/07). Peripheral blood mononuclear cells (PBMCs) were isolated by Histopaque-1077 density gradient centrifugation and monocytes were obtained by adhesion.

### 4.11. Analysis of Monocyte Adhesion

HBMECs or HBMEC-ACE2 were plated on coverslips previously treated with poly-L-lysine (Thermo Fisher Scientific Inc.) and incubated overnight to allow adhesion. Primary human monocytes were isolated and stained with CFSE (Thermo Fisher Scientific Inc.) and, at 24 hpi, these cells were added to HBMEC cultures, at a proportion of 20:1. After 30 min, the cultures were fixed with 4% formaldehyde, stained with DAPI, and analyzed using a ZEISS AxioImager D2 microscope. The number of DAPI- and CFSE-positive cells were counted using five different fields, totaling approximately 300 cells per experimental condition. The number of CFSE-positive monocytes per 100 HBMEC cells was calculated.

### 4.12. Evaluation of Endothelial Permeability and of Virus and PBMC Extravasation

HBMECs were seeded onto transwell inserts (Corning Costar, Kennebunk, ME, USA; 0.4 or 5 µm membrane) and confluence was monitored daily by measuring the transendothelial electrical resistance (TEER) across the cell monolayers using a Voltohmmeter (Millicell ERS-2). Once TEER reached high resistance (>80 Ω/cm^2^) [48], the cells were transduced with P-ACE2. TEER was measured again after transduction to confirm that it was not affected, with all wells displaying similar resistance values. The cells were then infected with SARS-CoV-2 as previously described. After 48 and 72 hpi, culture medium from the upper and lower chambers was collected, and the cells were incubated with FITC-conjugated BSA for 30 min. BSA extravasation was assessed by measuring fluorescence intensity in the lower chamber using a SpectraMax i3 spectrophotometer. In some assays, at 24 hpi, the cells were stimulated with DABK (1 µM) with or without a 30 min pretreatment with the B1R antagonist R954 (Ac-Orn-[Oic2, αMe Phe5, d-β Nal7, Ile8]des-Arg9-bradykinin; 1 µM) [75]. Blank inserts (without cells) were used to measure maximum fluorescence (100%) and to normalize experimental conditions. Infectious virus particles were measured in the upper and lower chambers by plaque assay. To evaluate cell transmigration, PBMCs (10:1 PBMCs/HBMEC) were added to the HBMEC cultures, and the number of cells in the lower chambers was determined by counting viable cells in a Neubauer chamber. Blank inserts were used to measure maximum PBMC extravasation (100%) and to normalize the experimental conditions.

### 4.13. Analysis of Angiotensin II Degradation by ELISA

HBMECs or HBMEC-ACE2 were cultured with exogenous Ang II (1 nmol), in the presence or absence of MLN-4760 (10^−6^ M, added 1 h before Ang II). After 6 h, Ang II concentration in the culture medium was measured using an Angiotensin II EIA Kit according to the manufacturer’s protocol (Sigma-Aldrich; Ref.: RAB0010). The MLN-sensitive Ang II degradation was calculated as the ratio between cultures with and without the inhibitor.

### 4.14. Statistical Analysis

Data were analyzed using the GraphPad Prism software v.8.4.0. Comparisons among every two groups were performed by *t*-test, and two-way ANOVA was used when different time points were considered. Comparisons including three or more groups were performed by one-way ANOVA, followed by Tukey’s post hoc test. Specific tests are indicated in the figure legends; *p* < 0.05 was considered statistically significant.

## 5. Conclusions

Taken together, our findings confirm that endothelial cells from the BBB can support SARS-CoV-2 replication, provided that ACE2 is expressed—a condition previously reported in human tissue samples but not reproducible in standard in vitro culture. By establishing an in vitro model permissive to infection, our study reinforces the central role of ACE2 in viral replication and enables a detailed analysis of SARS-CoV-2-induced endothelial activation and permeability. Notably, we demonstrated that ACE2 downregulation induced by SARS-CoV-2 infection potentiates HBMEC responsiveness to DABK, a product of the KKS—a pathway which is increasingly implicated in COVID-19 pathogenesis. This model provides a unique platform to explore the interaction between viral infection and KKS activation at the BBB interface. Future studies investigating the signaling pathways underlying DABK generation and response, as well as in vivo validation, will serve as proof of concept for clinical testing of therapeutic strategies targeting the KKS, particularly the B1 receptor.

## Figures and Tables

**Table 1:** Primers and probe sequences used for RT-qPCR assay.

Gene		Primer Sequence (5′–3′)
**N genomic**	Forward	TTACAAACATTGGCCGCAAA
**N genomic**	Reverse	GCGCGACATTCCGAAGAA
**N subgenomic**	Forward	CGATCTCTTGTAGATCTGTTCTCTAAACGAACAAATTAAAT
**N subgenomic**	Reverse	TCTGGTTACTGCCAGTTGCCTCTG
**Gapdh**	Forward	GTGGACCTGACCTGCCGTCT
**Gapdh**	Reverse	GGAGGAGTGGGTGTCGCTGT
**Ace2**	Forward	GGGATCAGAGATCGGAAGAAGAAA
**Ace2**	Reverse	AGGAGGTCTGAACATCATCAGT
**Ccl5**	Forward	CCAGCAGTCGTCTTTGTCAC
**Ccl5**	Reverse	CTCTGGGTTGGCACACACTT
**Ccl2**	Forward	CAGCCAGATGCAATCAATGCC
**Ccl2**	Reverse	TGGAATCCTGAACCCACTTCT
**Il8**	Forward	CAGCCAAAACTCCACAGTCA
**Il8**	Reverse	TTGGAGAGCACATAAAAACATCT
**Il6**	Forward	TGTGAAAGCAGCAAAGAGGCACTG
**Il6**	Reverse	ACAGCTCTGGCTTGTTCCTCACTA
**Tnf**	Forward	CAGAGGGAAGAGTTCCCCAGGGACC
**Tnf**	Reverse	CCTTGGTCTGGTAGGAGACGGC
**Gene**		**Probe (5′–3′)**
**N**		ACACTAGCCATCCTTACTGCGCTTCG

## Data Availability

The data that support the findings of this study are available in the Section 2 and Section 4, and/or Supplemental Material of this article.

## Supplementary Materials

The following supporting information can be downloaded at: https://www.mdpi.com/article/10.3390/ijms26125540/s1.
