# Supplementary material for: SARS-Cov-2 Replication in a Blood–Brain Barrier Model Established with Human Brain Microvascular Endothelial Cells Induces Permeability and Disables ACE2-Dependent Regulation of Bradykinin B1 Receptor
Source: Int J Mol Sci. 2025 Jun 10;26(12):5540. doi: 10.3390/ijms26125540 (PMC12193337; doi:10.3390/ijms26125540)
Supplement: Supplementary file 1 [file ijms-26-05540-s001.zip › ijms-3544523-supplementary.pdf]

Figure S1: Inflammatory stimuli do not potentiate the permissiveness of HBMECs to SARS-CoV-2

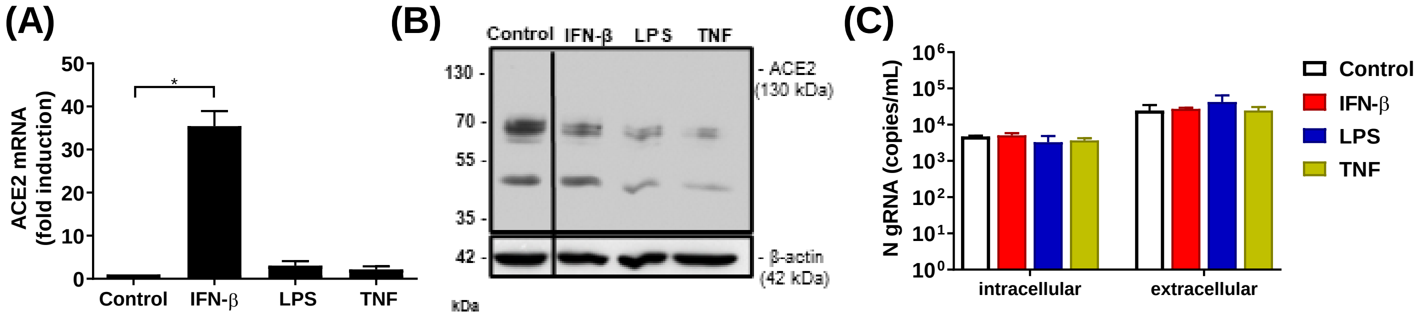

Figure S1: SARS-CoV-2 infection in immortalized HBMECs was not modulated by previous cell priming. **A**-**B**) HBMECs were cultured with IFN-β (1000 U) for 24h, LPS (1 µg/mL) for 4 h or TNF (10 ng/mL) for 4 h, and the expression of ACE2 mRNA and protein were analyzed by RT-qPCR (A) and western blotting (**B**), respectively. **C**) The cells were stimulated as in A and then infected with SARS-CoV-2 (A2; MOI=0.1). Viral genomic RNA was measured in the cell lysates (intracellular) and culture medium (extracellular) by RT-qPCR.

Figure S1

Figure S2: Time-course analysis of cell viability in HBMECs following SARS-CoV-2 infection, assessed by ATP-based luminescence assay

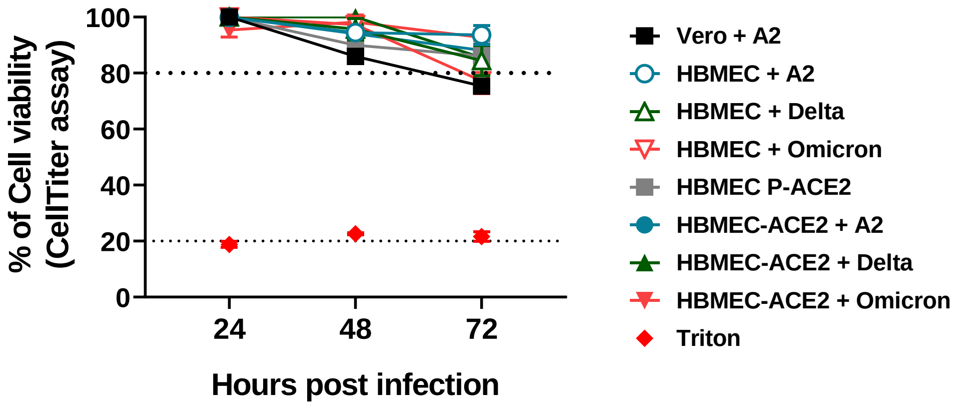

Figure S2: Time-course analysis of cell viability in HBMECs following SARS-CoV-2 infection, assessed by ATP-based luminescence assay. HBMECs and Vero cells were infected with the indicated strains of SARS-CoV-2, and cell viability was measured at the indicated time points post-infection by quantification of intracellular ATP levels using CellTiter Aqueous One Solution; 1% Triton X-100 treatment was used as a positive control for cytotoxicity. Data represent mean ± SD of triplicates from two independent experiments.

Figure S2

Figure S3: TMPRSS2 inhibitor reduces cytokine expression in SARS-CoV-2 infected HBMECs

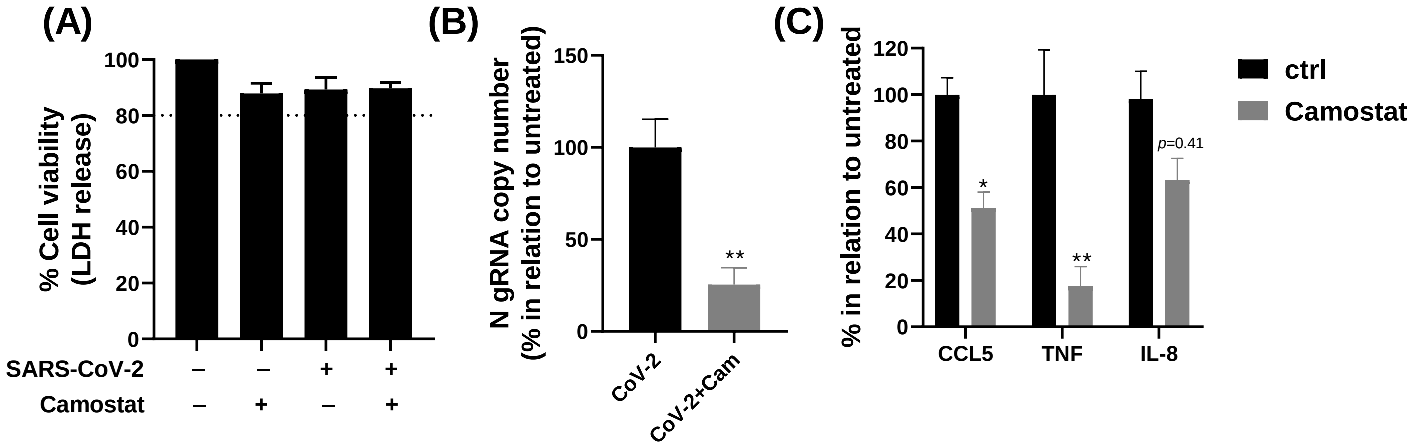

Figure S3: TMPRSS2 inhibitor reduces cytokine expression in SARS-CoV-2 infected HBMECs. HBMECs were treated with camostat mesylate, for 2h, and then infected with SARS-CoV-2 Delta strain (MOI=0.1). After 24h, cell viability was estimated by LDH activity in the culture medium (**A**); viral RNA was measured in the culture medium (**B**) and CCL5, TNF, and IL-8 expression were evaluated in the cell lysates by RT-qPCR (**C**). Data represent mean ± SD of triplicates from two independent experiments. *indicates $p<0.05$; **$p<0.01$

Figure S3
